# Microglia P2Y_6_ receptors mediate nitric oxide release and astrocyte apoptosis

**DOI:** 10.1186/s12974-014-0141-3

**Published:** 2014-09-03

**Authors:** Clara Quintas, Diana Pinho, Clara Pereira, Lucília Saraiva, Jorge Gonçalves, Glória Queiroz

**Affiliations:** Department of Drug Sciences, Laboratory of Pharmacology, REQUIMTE and Center for Drug Discovery and Innovative Medicines, Faculty of Pharmacy, University of Porto, Rua Jorge Viterbo Ferreira n° 228, Porto, 4050-313 Portugal; Department of Biological Sciences, Laboratory of Microbiology, REQUIMTE and Center for Drug Discovery and Innovative Medicines, Faculty of Pharmacy, University of Porto, Rua Jorge Viterbo Ferreira n° 228, Porto, 4050-313 Portugal; Present address: IBMC, Instituto de Biologia Molecular e Celular, Rua do Campo Alegre 823, Porto, 4150-180 Portugal

**Keywords:** lipopolysaccharide, astroglial proliferation, microglia, uracil nucleotides, P2Y_6_ receptors, nitric oxide, apoptosis

## Abstract

**Background:**

During cerebral inflammation uracil nucleotides leak to the extracellular medium and activate glial pyrimidine receptors contributing to the development of a reactive phenotype. Chronically activated microglia acquire an anti-inflammatory phenotype that favors neuronal differentiation, but the impact of these microglia on astrogliosis is unknown. We investigated the contribution of pyrimidine receptors to microglia-astrocyte signaling in a chronic model of inflammation and its impact on astrogliosis.

**Methods:**

Co-cultures of astrocytes and microglia were chronically treated with lipopolysaccharide (LPS) and incubated with uracil nucleotides for 48 h. The effect of nucleotides was evaluated in methyl-[^3^H]-thymidine incorporation. Western blot and immunofluorescence was performed to detect the expression of P2Y_6_ receptors and the inducible form of nitric oxide synthase (iNOS). Nitric oxide (NO) release was quantified through Griess reaction. Cell death was also investigated by the LDH assay and by the TUNEL assay or Hoechst 33258 staining.

**Results:**

UTP, UDP (0.001 to 1 mM) or PSB 0474 (0.01 to 10 μM) inhibited cell proliferation up to 43 ± 2% (n = 10, *P* <0.05), an effect prevented by the selective P2Y_6_ receptor antagonist MRS 2578 (1 μM). UTP was rapidly metabolized into UDP, which had a longer half-life. The inhibitory effect of UDP (1 mM) was abolished by phospholipase C (PLC), protein kinase C (PKC) and nitric oxide synthase (NOS) inhibitors. Both UDP (1 mM) and PSB 0474 (10 μM) increased NO release up to 199 ± 20% (n = 4, *P* <0.05), an effect dependent on P2Y_6_ receptors-PLC-PKC pathway activation, indicating that this pathway mediates NO release. Western blot and immunocytochemistry analysis indicated that P2Y_6_ receptors were expressed in the cultures being mainly localized in microglia. Moreover, the expression of iNOS was mainly observed in microglia and was upregulated by UDP (1 mM) or PSB 0474 (10 μM). UDP-mediated NO release induced apoptosis in astrocytes, but not in microglia.

**Conclusions:**

In LPS treated co-cultures of astrocytes and microglia, UTP is rapidly converted into UDP, which activates P2Y_6_ receptors inducing the release of NO by microglia that causes astrocyte apoptosis, thus controlling their rate of proliferation and preventing an excessive astrogliosis.

## Background

Chronic inflammation is characteristic of several brain disorders leading to loss of cognitive function. In the central nervous system (CNS), the inflammatory response is mediated by glial cells that acquire reactive phenotypes to participate in neuronal repair mechanisms [1,2]. In particular, astrocytes respond with a complex reaction named astrogliosis that includes several morphological and functional changes, such as cell hypertrophy, glial fibrillary acidic protein (GFAP) and nestin up-regulation [3], and cell proliferation [4]. These progressive changes are time and context dependent, being regulated by inflammatory mediators produced in the lesion site [5]. Activated microglia are the main source of these inflammatory mediators, assuming an important role in the modulation of astrogliosis progression during the course of the inflammatory response [6,7]. These mediators may be pro-inflammatory, such as IL-1β, TNF-α and nitric oxide (NO), or anti-inflammatory, such as IL-10, IL-4, TGF-β, according to the microglia phenotype, which is highly dependent on the pathological context [2,8]. Lipopolysaccharide (LPS) is an agonist of toll-like receptors-4 (TLR4), inducing a pro-inflammatory phenotype in microglia. However, chronic activation of TLR4 receptors has been shown to promote microglia polarization toward an anti-inflammatory phenotype [9,10], but its impact in the inflammatory response and in the modulation of astrogliosis remains to be established. In fact, different extents of astrogliosis and microgliosis have different impacts in neuronal regeneration [1,2]. In the extreme end of the astrogliosis spectrum, proliferating astrocytes may interact with fibroblasts and other glial cells to form a glial scar, creating an environment that prevents axon regeneration [11], leading to the idea that inhibition or control of this response would be beneficial to neuronal survival after injury. Therefore, the mediators produced by chronically activated microglia may have an important role to prevent excessive astrogliosis and promote neuronal regeneration and sprouting.

In a context of chronic brain inflammation, both adenine and uracil nucleotides attain high concentrations in the extracellular medium (in the mM range) due to cell damage or death, and activate P2 receptors in both types of glial cells, contributing to astrogliosis [12] and reinforcing the release of inflammatory messengers produced by microglia [13]. Particularly, the uracil nucleotides may activate pyrimidine receptors, such as the P2Y_2,4,6_ and P2Y_14_ receptor subtypes [14] that participate in the inflammatory response [15]. P2Y_6_ receptors contribute to the clearance of necrotic cell debris by stimulating microglia phagocytosis of dying neurons [16], whereas the P2Y_2_ receptors mediate astrocyte migration [17], but the effect of uracil nucleotides in the modulation of astroglial proliferation and their role in the control of glial scar formation is largely unknown.

To investigate the role of pyrimidine receptors in microglia-astrocyte signaling and its impact in the control of astrogliosis, it was used a cell culture model that could represent a state of chronic brain inflammation, which consisted of co-cultures of astrocytes and microglia submitted to a long-term treatment with LPS (0.1 μg/ml). The cultures obtained were used to investigate: i) the effect of uracil nucleotides in cell proliferation, ii) the influence of ectonucleotidases on uracil nucleotides metabolism and consequent impact in cell proliferation, iii) the signaling pathways and the mechanisms activated by the pyrimidine receptors involved in the control of cell proliferation, and iv) the contribution of microglia pyrimidine receptors to the modulation of astroglial proliferation.

## Methods

### Materials

The antibodies used and the respective information are listed in Table 1. The following drugs and reagents were used: L-arginine (L-ARG), lipopolysaccharide from *Salmonella thyphimurium* (LPS), N-nitro-L-arginine methyl ester hydrochloride (L-NAME), pertussis toxin (PTX), bisindolylmaleimide XI hydrochloride (RO 32-0432), penicillin, streptomycin, uracil, uridine, uridine-5’-monophosphate disodium (UMP), uridine-5’-diphosphate sodium (UDP), uridine 5'-triphosphate trisodium (UTP), uridine 5'-diphosphoglucose disodium (UDP-glucose), 1-[6-[((17β)-3-methoxyestra-1,3,5[10]-trien-17-yl)amino]hexyl]-2,5-pyrrolidinedione (U 73343), 1-[6-[((17β)-3-methoxyestra-1,3,5[10]-trien-17-yl)amino]hexyl]-1H-pyrrole-2,5dione (U 73122), 2'-(4-hydroxyphenyl)-5-(4-methyl-1-piperazinyl)-2,5'-bi-1H-benzimidazole trihydrochloride hydrate (Hoechst 33258), Ribonuclease A (RNAse) and propidium iodide (PI) from Sigma-Aldrich (Sintra, Portugal); N,N''-1,4 butanediylbis[N'-(3-isothiocyanatophenyl)thiourea] (MRS 2578) and 3-(2-oxo-2-phenylethyl)uridine-5'-diphosphate disodium (PSB 0474) from Tocris (Bristol, UK); methyl-[^3^H]thymidine (specific activity 80 to 86 Ci/mmol) and enhanced chemiluminescence (ECL) western blotting system from Amersham Biosciences (Lisbon, Portugal). Stock solutions of drugs were prepared with dimethyl sulfoxide or distilled water and kept at -20°C. Solutions of drugs were prepared from stock solutions diluted in culture medium immediately before use.

**Table 1:** **Primary and secondary antibodies used in immunocytochemistry and western blotting**

**Primary antibodies**				
**Antigen**	**Code**	**Host**	**Dilution**	**Supplier**
GFAP	G9269	Rabbit	1:600 (IF)	Sigma
GFAP	G6171	Mouse	1:600 (IF)	Sigma
CD11b	sc-53086	Mouse	1:50 (IF)	Santa Cruz Biotechnology, Inc
P2Y_6_	APR-011	Rabbit	1:200 (IF)	Alomone
			1:300 (WB)	
iNOS	AB5382	Rabbit	1:5 000 (IF)	Chemicon
Actin	sc-1615-R	Rabbit	1:200 (WB)	Santa Cruz Biotechnology, Inc
**Secondary antibodies**				
**Antigen**	**Code**	**Host**	**Dilution**	**Supplier**
TRITC anti-rabbit	T6778	Goat	1:400 (IF; GFAP, P2Y_6_)	Sigma
			1:2 000 (IF; iNOS)	
Alexa Fluor 488 anti-mouse	A-11034	Goat	1:400 (IF)	Mol. Probes
anti-rabbit conjugated to horseradish peroxidase	sc-2004	Goat	1:10 000 (WB)	Santa Cruz Biotechnology, Inc

IF, immunofluorescence; WB, western blot analysis.

### Cell cultures

Animal handling and experiments were in accordance with the guidelines prepared by Committee on Care and Use of Laboratory Animal Resources (National Research Council, USA), followed the Directive 2010/63/EU of the European Parliament and the Council of the European Union and were approved by the ethics committee of the Faculty of Pharmacy from the University of Porto. Primary co-cultures of astrocytes and microglia were prepared from newborn (P0-P2) Wistar rats (Charles River, Barcelona, Spain) as previously described [18] with minor modifications. Cell cultures were treated with 0.1 μg/ml LPS and were incubated at 37°C in a humidified atmosphere of 95% air, 5% CO2. The medium containing 0.1 μg/ml LPS was replaced one day after cell cultures preparation, and subsequently, twice a week, with LPS remaining in the cultures from the first day *in vitro* (DIV1) until the end of the experiments. Cultures were synchronized to a quiescent phase of the cell cycle, by shifting fetal bovine serum concentration in the medium from 10% to 0.1% for 48 h, and then used in experiments at DIV30.

### Immunocytochemistry

Cultures were fixed and permeabilized as described in previous studies [19]. For double immunofluorescence, cultures were incubated with the primary antibodies (Table 1) overnight at 4°C. Visualization of GFAP, CD11b, and P2Y_6_ receptors and iNOS positive cells was accomplished upon 1 h incubation, at room temperature, with the secondary antibodies (Table 1). In negative controls, the primary antibody was omitted. Cell nuclei were labeled with Hoechst 33258 (5 μg/ml) for 30 min at room temperature. To evaluate the percentage of microglia in the cultures, approximately 200 cells per culture were counted, and the number of CD11b positive cells was expressed as percentage of the total number of cells counted.

### DNA synthesis

Cultures grown in 24-well plates were incubated with uracil nucleotides or solvent for 48 h, and methyl-[^3^H]-thymidine was added in the last 24 h, at a concentration of 1 μCi/ml. Antagonists or enzymatic inhibitors were added to the medium 1 h before uracil nucleotides. In experiments performed in the presence of PTX, the drug was added to the culture medium 24 h before the uracil nucleotides. At the end of the 48-h period of incubation, the protein content and methyl-[^3^H]-thymidine incorporation were evaluated as previously described [20].

### Metabolism of nucleotides

The metabolism of nucleotides was evaluated as previously described [20]. Briefly, cultures were incubated with uracil nucleotides, all at 0.1 mM, and samples were collected at 0, 1, 3, 8, 24 and 48 h. For evaluation of UTP half-life, additional samples were collected at 0, 5, 10, 15, 30, 60 min. The uracil nucleotides or their metabolites were separated and quantified by ion-pair-reverse-phase high-performance liquid chromatography (HPLC) with UV detection set at 254 nm [21]. Standards were analyzed in the same conditions and the retention time identified was (min): uracil (0.95), uridine (1.32), UMP (2.15), UDP (4.40) and UTP (6.40). The concentration of nucleotides and metabolites was calculated by peak area integration, followed by interpolation in calibration curves obtained with standards.

### Western blot analysis

The expression of P2Y_6_ receptors was evaluated as previously described [22]. Membranes were probed for 2 h at room temperature with appropriately diluted primary rabbit polyclonal antibodies anti-P2Y_6_ or anti-actin, followed by the secondary antibody goat anti-rabbit IgG conjugated to horseradish peroxidase (Table 1). The immunocomplexes were detected by ECL.

### Nitric oxide assay

Cultures were incubated with uracil nucleotides or solvent for 48 h. The P2Y_6_ antagonist MRS 2578 or other enzyme inhibitors, when tested, were added 1 h before the uracil nucleotides. At the end of the 48-h period of incubation, the nitric oxide released into the culture medium was assessed by measuring the accumulation of nitrates plus nitrites according to the instructions of a Nitrate/Nitrite Colorimetric Assay kit (Cayman, France). The content of nitrates plus nitrites present in the supernatants was expressed as percentage of respective control.

### Cell cycle

The ability of uracil nucleotides to arrest glial cells in a specific cell cycle stage was evaluated in cultures treated with uracil nucleotides or solvent for 48 h. Cells were harvested by trypsinization, rinsed with ice-cold PBS and fixed in ice-cold 70% ethanol for 15 min at -20°C. Cells were rinsed again with PBS and incubated with 0.2 mg/ml RNAse A at 37°C for 15 min and further with 0.5 mg/ml propidium iodide for at least 30 min in the dark at room temperature. The percentage of cells in each phase of the cell cycle was determined by a flow cytometric analysis using the FACSCalibur flow cytometer from BD Biosciences (Enzifarma, Porto, Portugal) and the CellQuest software from BD Biosciences (Enzifarma, Porto, Portugal). Cell cycle phases were identified and quantified using ModFit LT software (Verity Software House Inc., Topsham, USA).

### Cell death assays

Necrotic cell death was assessed by measuring the lactate dehydrogenase (LDH) release with an enzymatic assay according to the manufacturer’s instructions (Sigma-Aldrich, Sintra, Portugal). Cultures were incubated with uracil nucleotides or solvent for 48 h. LDH activity was determined in the culture supernatants and respective extracts. The amount of LDH released into the culture medium was expressed as the percentage of total LDH.

Apoptotic cell death was evaluated either by the indirect terminal transferase-mediated dUTP-digoxigenin nick end-labeling (TUNEL) to detect DNA fragmentation using an ApopTag peroxidase detection kit (Millipore, Madrid, Spain), or by the analysis of nuclear morphology with Hoechst 33258 staining (described above). Cultures were treated with uracil nucleotides or solvent for 48 h and, when present, L-NAME was added 1 h before nucleotides. The number of TUNEL positive cells was evaluated as previously described [20]. The number of apoptotic cells, observed with Hoechst 33258 staining, was evaluated by analyzing eight high-power fields (×400) in each culture, and the number of cells showing shrunken nuclei with a bright fluorescence appearance was expressed as percentage of total cell number counted.

### Statistical analysis

Data are expressed as means ± standard errors of the mean (SEM) from n number of experiments. Statistical analysis was carried out using the unpaired Student’s t-test or ANOVA followed by Dunnett’s multiple comparison test. Significant differences were indicated by *P* values lower than 0.05.

## Results

### Characterization of the co-cultures

**Figure 1:**
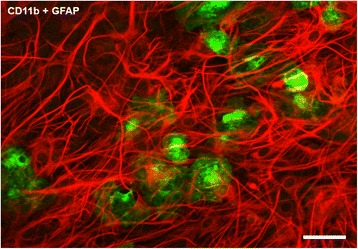
**Immunofluorescent micrograph representative of co-cultures treated with lipopolysaccharide (LPS) (0.1 μg/ml).** Astrocytes were labeled with rabbit anti-GFAP (TRICT, red) and microglia with mouse anti-CD11b (Alexa Fluor 488, green). The percentage of microglia in cultures was 4.36 ± 0.42% (n = 5). Scale bar: 50 μm.

The primary cortical brain cultures treated with lipopolysaccharide (LPS; 0.1 μg/ml) for 30 days *in vitro*, consisted of monolayers of astrocytes exhibiting a flattened, polygonal morphology and containing 4.36 ± 0.42% (n = 5) of microglia spread over the top of the astrocyte monolayer (Figure 1). The co-cultures obtained were named LPS cultures where microglial cells exhibited an amoeboid phenotype with retracted or short thick processes, suggestive of their activation [23], as expected for *in vitro* LPS treated microglia. In support of the potential of LPS to activate microglia and to prevent their proliferation, it was observed that in co-cultures grown without any treatment, the percentage of microglia was higher, approximately 8.0%, and presented longer processes [22].

In this study, LPS cultures were used to study the effect of uracil nucleotides in cell proliferation, as well as the contribution of activated microglia to this response.

### Effects of uracil nucleotides in cell proliferation

**Figure 2:**
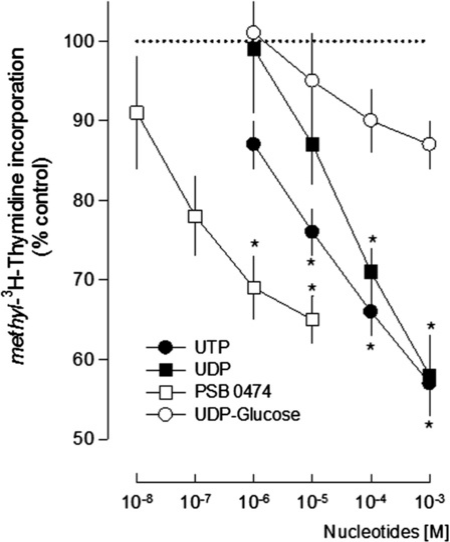
**Effects of uracil nucleotides in cell proliferation.** Lipopolysaccharide (LPS) cultures were incubated with nucleotides for 48 h and in the last 24 h methyl-[^3^H]-thymidine was added to the medium at a concentration of 1 μCi/ml. Effects in cell proliferation were estimated by methyl-[^3^H]-thymidine incorporation and expressed in percentage of control. Values are means ± SEM from five to ten experiments. *P <0.05, significant differences from control.

LPS cultures were incubated with several uracil nucleotides to evaluate their influence in cell proliferation. UTP that activates the P2Y_2,4_ subtypes, UDP and its analogue PSB 0474, both selective for the P2Y_6_ receptors and UDP-glucose that is selective for the P2Y_14_ receptors [14] were tested in a wide range of concentrations. Except for UDP-glucose, the uracil nucleotides UTP, UDP and PSB 0474 caused a concentration-dependent inhibition of cell proliferation (Figure 2).

### Extracellular metabolism of uracil nucleotides

**Figure 3:**
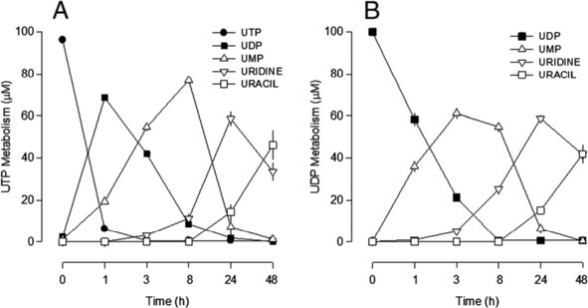
**Metabolism of uracil nucleotides in lipopolysaccharide (LPS) cultures.** Cells were incubated with 0.1 mM of **(A)** UTP or **(B)** UDP and samples were collected at 0, 1, 3, 8, 24 and 48 h. Uracil nucleotides and their metabolites were quantified by HPLC-UV. Values are means ± SEM from four experiments.

UTP metabolism was very fast, with a half-life of 10.3 ± 0.5 min (n = 5), and the main metabolite formed during the first hour was UDP, which remained in the medium up to 8 h (Figure 3A). When tested from the beginning, UDP metabolism was much slower compared to that of UTP (Figure 3B); its half-life was 77.3 ± 2.3 min (n = 4; *P* <0.05). The PSB 0474 half-life could not be evaluated because the highest concentration tested that caused an inhibition of cell proliferation was still below the detection limit of the method used to study the metabolism of these compounds.

### Expression and pharmacological characterization of the P2Y receptor subtype involved in the inhibition of cell proliferation induced by uracil nucleotides

**Figure 4:**
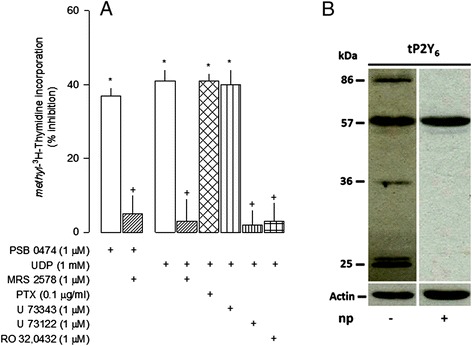
**Pyrimidine receptors and signaling pathway involved in the inhibition of cell proliferation mediated by uracil nucleotides. (A)** Lipopolysaccharide (LPS) cultures were incubated with the nucleotides for 48 h and in the last 24 h methyl-[^3^H]-thymidine was added to the medium at a concentration of 1 μCi/ml. The P2Y_6_ antagonist MRS 2578 and enzyme inhibitors were added to the medium 1 h before the nucleotides, except PTX, which was added to the medium 24 h before. Effects in cell proliferation were estimated by methyl-[^3^H]-thymidine incorporation and expressed as percentage of change from the respective control. Values are means ± SEM from eight to twenty experiments. *P <0.05, significant differences from respective control; +*P* <0.05, significant differences from the agonist alone. **(B)** Representative western blots showing the expression of P2Y_6_ receptors obtained from whole cell lysates. Two bands of 25 kDa, one of 36 kDa and another of 86 kDa, specifically reacted with the anti-P2Y_6_ antibody. These bands were absent in the presence of the respective neutralizing peptide (np).

The inhibitory effect of both PSB 0474 (1 μM) and UDP (1 mM) in cell proliferation was abolished by the selective P2Y_6_ receptor antagonist MRS 2578 (1 μM; Figure 4A). The inhibitory effect of UTP (0.1 mM) was also abolished by MRS 2578 (not shown), suggesting that this effect depends on its conversion into UDP and activation of P2Y_6_ receptors. Uncoupling G_i/o_ proteins from receptors with pertussis toxin (PTX, 0.1 μg/ml) did not change the effect of UDP (1 mM), which was attenuated by the phospholipase C (PLC) inhibitor U 73122 (1 μM), but not by its inactive analog U 73343 (1 μM), and by the protein kinase C (PKC) inhibitor RO 32-0432 (1 μM; Figure 4A), confirming the coupling of P2Y_6_ receptors to the Gq-PLC-PKC pathway.

**Figure 5:**
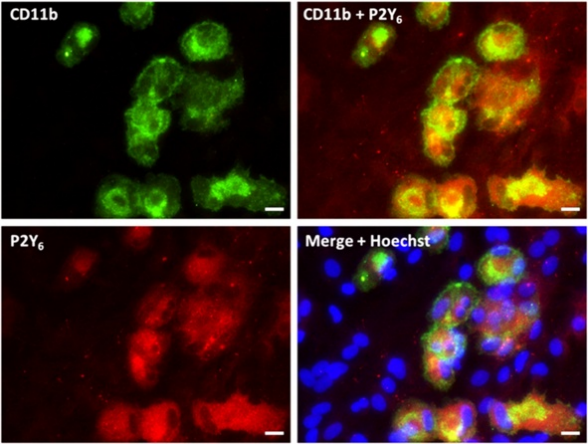
**Cellular distribution and localization of P2Y**_**6**_ **receptors in lipopolysaccharide (LPS) cultures.** Microglia were labeled with mouse anti-CD11b (Alexa Fluor 488, green), P2Y_6_ receptors were labeled with rabbit anti-P2Y_6_ (TRICT, red) and nuclei were labeled with Hoechst 33258 (blue). The orange spots represent the expression of P2Y_6_ receptors that are coincident with microglia, but not in astrocytes (blue nuclei that do not label with CD11b and P2Y_6_ receptor antibodies). Scale bar: 20 μm.

P2Y_6_ receptor expression in LPS cultures comprised four bands, two of 25 kDa, one of 36 kDa and another of 86 kDa, which were all absent in the presence of the P2Y_6_ receptor neutralizing peptide (np, Figure 4B). Analysis of the cellular localization of P2Y_6_ receptors by immunocytochemistry revealed a preferential co-localization with microglia (Figure 5), suggesting that uracil nucleotides may inhibit cell proliferation via microglial cells.

### P2Y_6_ receptor-mediated nitric oxide production

**Figure 6:**
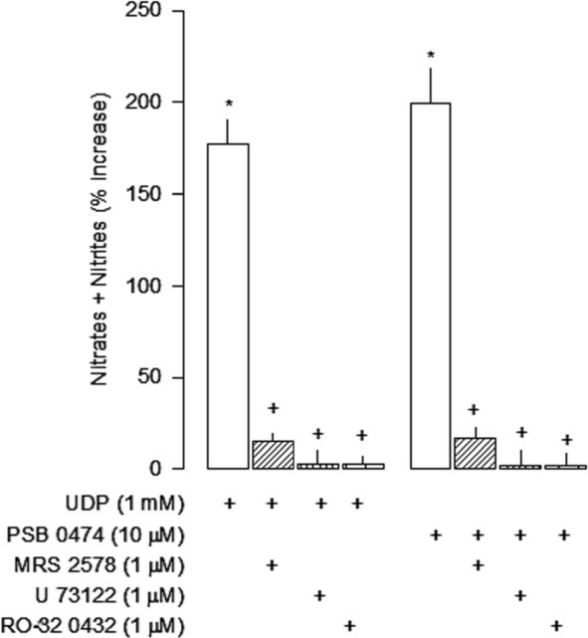
**Nitric oxide synthesis mediated by uracil nucleotides in lipopolysaccharide (LPS) cultures.** Cells were incubated with UDP or PSB 0474 for 48 h. The P2Y_6_ antagonist MRS 2578 and enzyme inhibitors were added to the medium 1 h before the nucleotides. The concentration of nitrites plus nitrates was evaluated in the culture supernatants and was expressed as percentage of change from the respective control. Values are means ± SEM from four experiments. *P <0.05, significant differences from the respective control; +*P* <0.05, significant differences from the agonist alone.

LPS increases iNOS expression and NO production by microglia [24,25], but this effect is attenuated during chronic LPS stimulation [9,10]. Since NO may inhibit astroglial proliferation [26] it was investigated whether P2Y_6_ receptor activation by uracil nucleotides modulated NO release in chronic LPS stimulated microglia. UDP (1 mM) and PSB 0474 (10 μM) increased NO release in the culture medium (Figure 6), an effect abolished by the selective antagonist of the P2Y_6_ receptors MRS 2578 (1 μM) and by the PLC inhibitor U 73122 (1 μM), or by the PKC inhibitor RO 32-0432 (1 μM; Figure 6). Additionally, the inhibitory effect of UDP in cell proliferation (1 mM; 44 ± 2, n = 25) was abolished by the NOS inhibitor L-NAME (0.1 mM; 7 ± 3, n = 8, *P* <0.05), and this effect was reversed in the presence of L-arginine (3 mM; 28 ± 6, n = 6; *P* <0.05).

**Figure 7:**
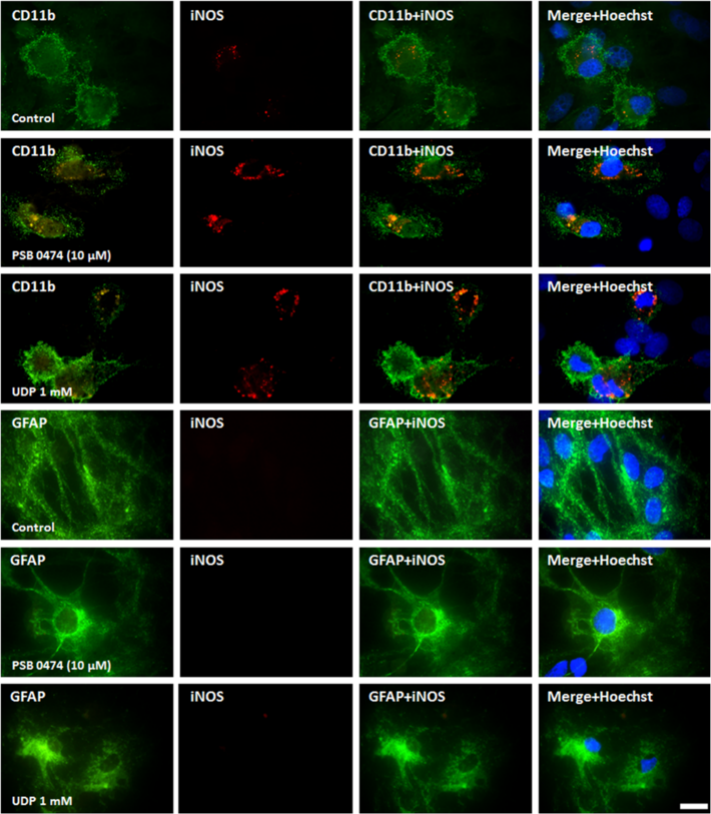
**Cellular localization of inducible nitric oxide synthase (iNOS) in lipopolysaccharide (LPS) cultures.** Cells were incubated with UDP or PSB 0474 for 48 h. Microglia were labeled with mouse anti-CD11b (Alexa Fluor 488, green), astrocytes with mouse anti-GFAP (Alexa Fluor 488, green) and iNOS with rabbit anti-iNOS (TRITC, red). Cell nuclei were labeled with Hoechst 33258 (blue). The orange spots represent the expression of iNOS in the cells and are coincident with an increased expression of iNOS in microglia, but not in astrocytes, upon stimulation with the uracil nucleotides. Scale bar = 10 μm.

In order to identify the cellular source of NO released upon P2Y_6_ receptor activation, the expression of iNOS was immunolocalized either with microglia or astrocytes in LPS cultures. No iNOS expression was detected in astrocytes, in control conditions and after treatment with the uracil nucleotides (Figure 7), whereas in microglia iNOS expression was residual in control conditions but was significantly increased after 48 h incubation with PSB 0474 (10 μM) or UDP (1 mM; Figure 7).

### P2Y_6_ receptor-mediated inhibition of cell proliferation: mechanisms involved

In these experimental conditions, microglial cells are responsible for NO production mediated by P2Y_6_ receptors. To clarify the mechanisms behind uracil nucleotides inhibition of cell proliferation, their effect on cell cycle progression and cell death was investigated.

Uracil nucleotides had no effect in cell cycle progression of glial cells. The percentage of cells in G_0_/G_1_, S or G_2_/M phase of the cell cycle was similar in control cultures (75.4 ± 1.8, 17.6 ± 3.2, 7.1 ± 1.5, respectively, n = 3) and those treated with UDP (1 mM; 76.4 ± 1.8, 13.6 ± 2.8, 10.0 ± 1.5, respectively, n = 3), therefore excluding cell cycle arrest as the mechanism involved in the inhibition of cell proliferation.

**Figure 8:**
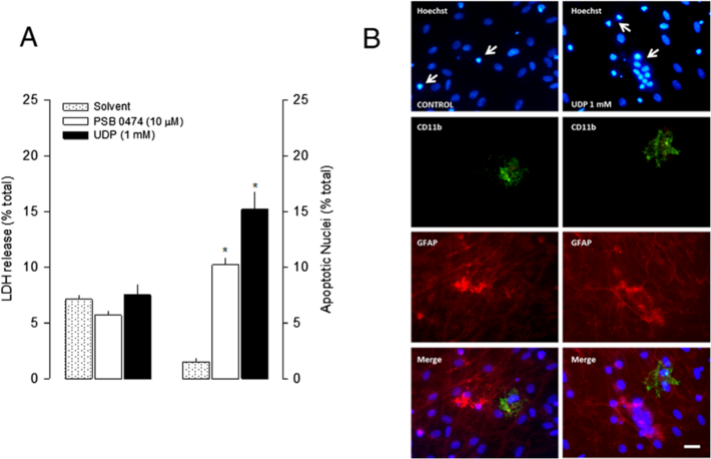
**Effects of uracil nucleotides in cell death in lipopolysaccharide (LPS) cultures. (A)** Necrotic cell death was evaluated by measuring the release of lactate dehydrogenase (LDH) and apoptotic cell death was evaluated by the TUNEL assay, after incubation with uracil nucleotides or solvent for 48 h. LDH activity was measured in the culture medium and in the culture extracts and the fraction released is represented in percentage of total LDH. The number of apoptotic cells was expressed as percentage of the total number of cells counted. Values are means ± SEM from four to seven experiments. *P <0.05, significant differences from respective control (solvent). **(B)** Cellular localization of apoptotic nuclei, obtained with the Hoechst 33258 staining in LPS cultures. Astrocytes were labeled with rabbit anti-GFAP (TRITC, red), microglia with mouse anti-CD11b (Alexa Fluor 488, green) and cell nuclei with Hoechst 33258 (blue). LPS cultures were incubated with solvent or UDP for 48 h. Shrunken nuclei with a bright fluorescence appearance, characteristic of apoptotic nuclei are clearly coincident with astrocytes (white arrows), but not with microglia. Scale bar = 20 μm.

Another possibility was that inhibition of cell proliferation mediated by uracil nucleotides could result from an increase in cell death. UDP (1 mM) and PSB 0474 (10 μM) caused no change in LDH release, which excluded cell death by necrosis (Figure 8A). However, both UDP (1 mM) and PSB 0474 (10 μM) induced cell death by apoptosis assessed by the TUNEL assay (Figure 8A).

Cultures treated with UDP (1 mM) showed an increase in the number of shrunken nuclei with a bright fluorescence appearance obtained by Hoechst 33258 staining (Figure 8B). The percentage of apoptotic nuclei in control cultures was 6.75 ± 0.65% (n = 8) and increased to 16.02 ± 0.75% (n = 8, *P* <0.05) in cultures treated with UDP (1 mM). This increase in the number of apoptotic cells was attenuated to 11.83 ± 0.61% (n = 8, *P* <0.05) when UDP was tested in the presence of L-NAME (0.1 mM). Additionally, the apoptotic nuclei co-localized with astrocytes but not with microglia (Figure 8B).

## Discussion

The CNS, with the contribution of glial cells (both astrocytes and microglia), has been shown to be able to mount an innate immune reaction in response to danger signals, such as endogenous nucleotides released upon cerebral injury or exogenous pathogens, systemic bacteria or virus [27]. It is now well recognized that microglia functional plasticity is strictly stimuli-dependent [2,8]; however, it is not known how microglia coordinate the inflammatory response and the progress of astrogliosis in a paradigm of chronic glia activation, characteristic of several inflammatory pathologies. Exposure of CNS to endotoxins, such as LPS, is a useful approach to activate immunity, in particular microglial cells [28]. Therefore, such immunological challenge was used in an *in vitro* model where microglia and astrocytes were present and could cooperate to reproduce some of the conditions that might be observed during chronic brain inflammation. It consisted of co-cultures of astrocytes containing 4 to 5% microglia chronically stimulated with 0.1 μg/ml LPS for 30 days.

In previous studies, we have shown that in co-cultures of astrocytes and microglia containing a higher percentage of microglia, but without LPS treatment, UTP caused an inhibition of cell proliferation that could be correlated with a higher expression of P2Y_6_ receptors in microglia. In contrast, in highly enriched astroglial cultures, either treated or not with LPS, uracil nucleotides had no effect in cell proliferation [22], suggesting a fundamental role of microglial cells to the P2Y_6_ receptor-mediated inhibitory effect. In LPS cultures, UTP also inhibited cell proliferation and this effect was extensive to UDP and to the selective agonist of the P2Y_6_ receptors PSB 0474 [29], but not to the selective agonist of P2Y_14_ receptors UDP-glucose [30]. The inhibitory effect of uracil nucleotides was mediated by P2Y_6_ receptors, since it was abolished by MRS 2578, the selective antagonist of this receptor subtype [31]. Although UTP has some affinity for P2Y_6_ receptors [32], its inhibitory effect was mainly dependent on its metabolism and formation of UDP, since UTP had a very short half-life, being rapidly converted into UDP, which remained in the culture medium for about 8 h. This conclusion is further supported by the observation that an inhibitory effect of UTP and of other uracil nucleotides was already observed within 8 h of incubation (not shown) before a significant accumulation of the other metabolites, such as uridine or uracil could be detected in the culture medium.

UDP half-life was much longer than that of UTP, which may be explained by the NTPDases expressed in these cultures. Microglia express mainly NTPDase1, which hydrolyses UTP faster than UDP [33], and astrocytes, the main cell type present in these cultures, express high levels of NTPDase2 [34]. This enzyme seems to be upregulated upon LPS stimulation [35] and preferentially hydrolyzes UTP compared to UDP [36]. Thus, the predominance of NTPDase2 activity in these cultures favors UDP accumulation and a preferential activation of P2Y_6_ receptors.

P2Y_6_ receptors were coupled to the Gq-PLC-PKC pathway [14]; however activation of this pathway in astrocytes has been shown to mediate cell proliferation [20], suggesting a microglial localization. Therefore, the mechanisms involved in the inhibitory effect mediated by P2Y_6_ receptors were further investigated by looking to the cellular localization of these receptors. Expression of P2Y_6_ receptors in LPS cultures revealed a multiple band pattern, as previously described and discussed [22]. Only three of the four bands were lost after adsorption with the neutralizing peptide, suggesting this antibody is detecting some other antigen [37]. The cellular localization of P2Y_6_ receptors was analyzed by immunocytochemistry, which revealed that this P2Y_6_ antibody reacts mainly with microglial antigens. This observation is also in agreement with previous studies showing an up-regulation of P2Y_6_ receptor expression in LPS-activated microglia [16,38]. Although there are no specific P2Y_6_ antibodies available [37], results obtained by western blot and immunocytochemistry analysis, together with pharmacological data and the fact that UDP had no effect in highly enriched astroglial cultures [22], all support the conclusion that P2Y_6_ receptors are mainly localized in microglia.

LPS increases iNOS expression and NO production in microglia via PKC activation [24,25,39]. However, NO release by microglia decays upon chronic LPS stimulation [9,10] suggesting a downregulation of this signaling pathway. In LPS cultures, all the uracil nucleotides that inhibited cell proliferation also increased the release of NO, an effect mediated by P2Y_6_ receptors coupled to the PLC-PKC pathway. Thus, a crosstalk between LPS and P2Y_6_ receptors signaling pathways may ensure additional iNOS expression and NO production when LPS response is already downregulated. The inhibitory effect of UDP was also prevented by L-NAME, a NOS inhibitor, confirming the involvement of NO that was shown to be exclusively produced by microglia iNOS [25,40]. Our results confirm previous observations, since only microglia showed iNOS immunoreactivity, which was upregulated when LPS cultures were treated with uracil nucleotides, suggesting that microglia are the main source of NO detected in the culture medium, either under basal conditions or upon P2Y_6_ receptor activation.

NO may potentially damage cells through the formation of reactive nitrogen species that cause DNA fragmentation [41]. This mechanism could be responsible for the inhibition of cell proliferation induced by uracil nucleotides, since these compounds increased the number of cells presenting DNA fragmentation, an indicator of cell death by apoptosis. In some systems NO-mediated apoptosis is preceded by cell cycle arrest [42], which was not observed in this study. In LPS cultures, UDP induced cell death without any previous effect in the cycle of confluent and synchronized cells. P2Y_6_ receptor activation and NO release only affected astrocytes, but not microglia viability. Shrunken nuclei with a bright fluorescence appearance, indicating chromatin condensation characteristic of apoptotic cells, were highly coincident with astrocytes but did not co-localize with microglia. Because cell population in the S-phase of the cell cycle was not changed by UDP, an increase in NO release seems to result from iNOS upregulation of individual pre-existent microglia, excluding the possibility of microglia proliferation. This result also indicates that P2Y_6_ receptors do not mediate microglia proliferation and therefore, their activation cannot modify the anti-proliferative profile established by 0.1 μg/ml LPS [43].

Although uracil nucleotides had no effect in highly enriched astroglial cultures, cell death by apoptosis mediated by pyrimidine receptors was already observed in co-cultures of astrocytes and microglia (without LPS treatment), but to a smaller extent [22], suggesting that LPS and/or uracil nucleotides are not able to induce astrocyte death without the contribution of microglia. Nevertheless, it seems that in co-cultures of astrocytes and microglia, LPS potentiates astrocyte apoptosis mediated by uracil nucleotides, since it increases from 6% in co-cultures without LPS treatment [22] to 15% in LPS cultures. LPS facilitates P2Y_6_ receptor-mediated NO release by microglia, but other cytokines such as IL-1β or TNF-α [44] may come into play contributing to astroglial cell death.

## Conclusions

**Figure 9:**
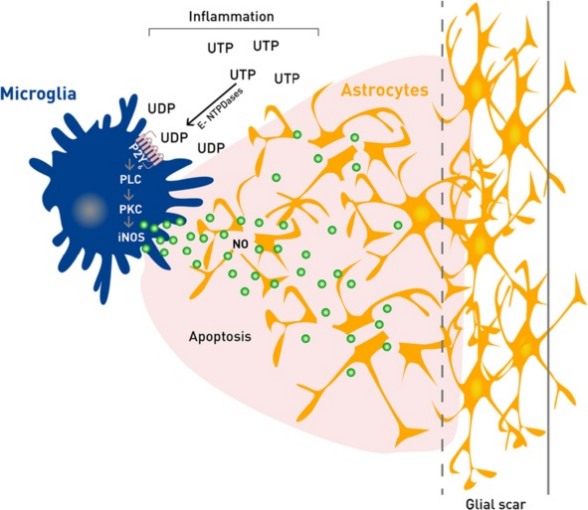
**Schematic representation of the purinergic mechanisms mediating microglia-astrocyte communication in lipopolysaccharide (LPS) cultures.** Uracil nucleotides released during inflammatory response activate microglia P2Y_6_ receptors coupled to the phospholipase C (PLC) - protein kinase C (PKC) pathway, which mediates an increase in inducible nitric oxide synthase (iNOS) expression and consequently, in nitric oxide (NO) release. Diffusible NO mediates astroglial apoptosis.

The present study shows that chronically activated microglia influence the astroglial response to uracil nucleotides favoring astroglial apoptosis as a consequence of microglial P2Y_6_ receptor activation that induces NO release (Figure 9). Therefore, P2Y_6_ receptor activation may represent an important mechanism by which microglia control excessive astrogliosis that may hamper neuronal regeneration. Nevertheless, it is known that human cortical astrocytes are diverse and structurally and functionally more complex than their rodent counterparts [45]; therefore this hypothesis should be further confirmed within human glial cells.

## Abbreviations

ECL: enhanced chemiluminescence
GFAP: glial fibrillary acidic protein
HPLC: high-performance liquid chromatography
iNOS: inducible form of nitric oxide synthase
LDH: lactate dehydrogenase
LPS: lipopolysaccharide
L-NAME: N-nitro-L-arginine methyl ester
MRS 2578: N,N''-1,4-butanediylbis[N'-(3isothiocyanatophenyl)thiourea]
NO: nitric oxide
NOS: nitric oxide synthase
NTPDases: nucleoside triphosphate diphosphohydrolases
PKC: protein kinase C
PLC: phospholipase C
PSB 0474: 3-(2-oxo-2-phenylethyl)uridine-5'-diphosphate
PTX: pertussis toxin
RO 32-0432: bisindolylmaleimide XI
TLR4: toll-like receptors-4
TRITC: tetramethylrodamine isothiocyanate
TUNEL: terminal transferase-mediated dUTP-digoxigenin nick end-labeling
U 73343: 1-[6-[((17β)-3methoxyestra-1,3,5[10]-trien-17-yl)amino]hexyl]-2,5-pyrrolidinedione
U 73122: 1-[6-[((17β)3-methoxyestra-1,3,5[10]-trien-17-yl)amino]hexyl]-1H-pyrrole-2,5-dione
